# 1-Octanol-assisted ultra-small volume droplet microfluidics with nanoelectrospray ionization mass spectrometry

**DOI:** 10.1016/j.aca.2024.342998

**Published:** 2024-07-21

**Authors:** Yaoyao Zhao, Insu Park, Stanislav S. Rubakhin, Rashid Bashir, Yurii Vlasov, Jonathan V. Sweedler

**Affiliations:** a Department of Chemistry and Beckman Institute for Advanced Science and Technology, University of Illinois Urbana–Champaign, Urbana, IL, 61801, USA; b Holonyak Micro & Nanotechnology Laboratory, University of Illinois Urbana–Champaign, Urbana, IL, 61801, USA; c Beckman Institute for Advanced Science and Technology, Holonyak Micro & Nanotechnology Laboratory, and Department of Electrical and Computer Engineering, University of Illinois Urbana–Champaign, Urbana, IL, 61801, USA; d Department of Bioengineering, University of Illinois at Urbana–Champaign, Urbana, IL, 61801, USA

**Keywords:** Cerebral spinal fluid, Neurotransmitters, Low volume sampling, Droplet microfluidics, Mass spectrometry

## Abstract

**Background:**

Droplet microfluidics with push-pull and microdialysis sampling from brain slices, cultured cells and engineered tissues produce low volume mass limited samples containing analytes sampled from the extracellular space. This sampling approach coupled to mass spectrometry (MS) detection allows evaluation of time-dependent chemical changes. Our goal is an approach for continuous sampling and segregation of extracellular samples into picoliter droplets followed by the characterization of the droplets using nanoelectrospray ionization (nESI) MS. The main focus here is the optimization of the carrier oil for the microfluidic device that neither affects the stability of picoliter droplets nor compatibility with MS detection of a range of analytes.

**Results:**

We developed and characterized a 1-octanol-assisted ultra-small volume droplet microfluidic nESI MS system for the analysis of neurotransmitters in distinct samples including cerebrospinal fluid (CSF). The use of a 1-octanol oil phase was effective for generation of aqueous droplets as small as 65 pL and enabled detection of acetylcholine (ACh) and gamma-aminobutyric acid (GABA) in water and artificial CSF. Continuous MS analysis of droplets for extended periods up to 220 min validated the long-term stability of droplet generation and analyte detection by nESI-MS. As an example, ACh response demonstrated a linear working range (R^2^ = 0.99) between 0.4 μM and 25 μM with a limit of detection of 370 nM (24 amol), enabling its quantitation in rodent CSF.

**Significance:**

The established droplet microfluidics – nESI MS approach allows the analysis of microenvironments at high spatiotemporal resolution. The approach may allow microsampling and monitoring of spatiotemporal dynamics of neurochemicals and drugs in the brain and spinal cord of live animals.

## GRAPHICAL ABSTRACT

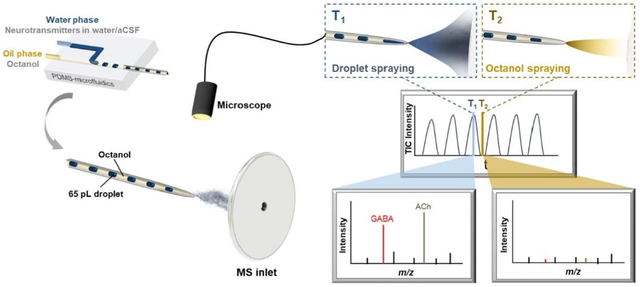

## 1. Introduction

Droplet-based microfluidics is an effective technology for biological fluid sampling and chemical characterization. Here two streams of immiscible solutions/phases are meet at the junction forming a stream of aqueous droplets of controlled volumes separated by plugs of carrier phase, usually oil [1–4]. The droplet-based platforms can be automated for high throughput droplet generation and analysis. With these advantages, droplet-based microfluidic devices have found a wide range of applications in various scientific disciplines [5–7]. For example, localized and continuous sampling of cerebrospinal fluid (CSF) from extracellular spaces via droplet microfluidic approaches improves temporal resolution of neurochemical analyses compared to standard continuous micro-dialysis and push-pull sampling techniques [8,9]. The digitization of samples avoids analyte diffusion between the droplets and allows longer times for sample storage and off-line sample analysis using a variety of measurement approaches [10,11]. A number of analyte detection approaches have been integrated with droplet microfluidics including electrochemistry [12], Raman spectroscopy [13], and mass spectrometry (MS) [14].

Mass spectrometry is one of most versatile analyte detection approaches integrated with droplet microfluidics [1,11,17–19] (summarized in recent reviews [15,16]). For example, nanoelectrospray ionization mass spectrometry (nESI-MS) is a multiplexed analytical technique exhibiting high sensitivity, extended analyte coverage, and accuracy in qualitative and quantitative analyses [20,21]. Compared with ESI-MS operating with larger volumes of spraying samples, nESI-MS presents improved analyte ionization efficiency and sample’s chemical matrix tolerance. nESI-MS is suitable for analyzing of small-volume chemically complex samples at high throughput and compatible with segmented flow systems such as droplet microfluidic devices [22]. Recent advances in integration of droplet-based microfluidics and nESI have been successfully utilized in high-throughput analysis of ultra-small (in pL and nL) droplets [2,22–25].

There are several challenges in the hyphenation of droplet microfluidics to MS detection. Among them, the carrier oil surrounding the segmented aqueous phase can impair electrospray plume stability [21, 26]. This includes oil accumulation at the ESI emitter’s tip affecting the Taylor cone formation for new aqueous droplets [4]. One approach to overcome this limitation has been to use an additional continuous aqueous stream mixing with content of aqueous droplets from segmented by oil stream. As result, uninterrupted Taylor cone is formed at the inlet of the mass spectrometer [27–29]. However, dilution droplet content can cause a decrease in sensitivity. Also, increase of inter-droplet intervals may be needed for reduction of droplet content overlap affecting performance of higher throughput analyses [30]. Another strategy to avoid analyte dilution involves the removal of the oil segments before MS. The Kennedy group reported the carrier oil with high viscosity such as FC-40 and PFD did not electrospray under the voltages optimal for electrospraying of aqueous droplets, thus enabling phase separation at the emitter tip [31].

Recently, there have been a number of advances in sensitive analysis of analytes using droplet microfluidics with both ESI-MS and MALDI-MS detections [4,18,19,21,25,32,33]. Kennedy’s group enabled stable nESI-MS analysis of droplet samples from 65 pL to 1.2 nL using PDMS chips with 2 % 008-fluorosurfactant in Novec 7500 as the oil phase [22]. We recently reported a silicon microfluidic platform with nESI emitters integrated with droplet generators that enables efficient phase separation at the nESI tip [23] and provides LOD at a level of a few attomoles in a droplet volume of 7 pL [24]. These devices can be used to interrogate the chemical composition of the fluid around living tissues in-vitro with high spatiotemporal resolution. The real time nature of the droplet microfluidic sample collection coupled with nESI-MS can enable investigation of biological models such as organoids and engineered tissues used as models of disease conditions such as inflammation.

Here we explore detection of pL-scale samples with a more traditional microfluidics platform based on polydimethylsiloxane (PDMS) interfaced with commercial nESI emitters. We demonstrate that 1-octanol, a non-ionic surfactant, works well as the organic phase that does not contaminate of mass spectrometer due to its volatile nature, improves the linearity of detection and quantification of neurotransmitters when using isotopically labeled internal standards in different media including CSF. Restricting the analyte to discrete droplets also enables long term storage of the samples before measurements [34,35]. This can be important when monitoring of the spatiotemporal dynamics of neurochemical levels in extracellular spaces of the brain allowing investigation of a variety of normal and pathological brain functions [36,37], even when collection and analysis are at distinct locations. We optimized the approach using the neurotransmitters acetylcholine (ACh) and γ-aminobutyric acid (GABA) as they have critical roles in formation of different behaviors, drug effects, or disease states [38,39] as well as being difficult to determine using electrochemical and fluorescence detection approaches. More specifically, our system generates 65 pL droplets using commercial syringe pumps attached to a microfluidic device. The system has the potential to be applied for *in vivo* monitoring of neurochemicals and infused drugs from brain slices, engineered tissues and the intact brain using *in vivo* microdialysis.

## 2. Experimental

### 2.1. Chemicals

Artificial cerebral spinal fluid (aCSF) was purchased from Tocris Bioscience (Bristol, UK). 1-octanol, ACh and GABA were acquired from Sigma Aldrich (St. Louis, MO). Aquapel came from Aquapel Glass Treatment (Cranberry Twp, PA, USA). ACh-d4 was produced by CDN isotopes (Pointe-Claire, Canada). All other reagents were purchased from Fisher Scientific. Chemicals were used as is without further purification.

### 2.2. Chip design and fabrication

Polydimethylsiloxane (PDMS) microfluidic devices were fabricated using a standard soft lithography technique with SU8–3050 photoresist (MicroChem Corp, Newton, MA). Width and depth of aqueous phase channel is 30 μm × 30 μm and carrier oil phase channel 50 μm × 30 μm, respectively. The inlet and outlet junctions were designed as a serration for mechanical fitting the fused silica glass capillary into PDMS to avoid solution leakage. PDMS and curing agent (10:1 mixing ratio) were mixed and poured on the master wafer and cured for 2 h at 60 °C. PDMS master was attached to flat PDMS slab by activating hydrophilic surface during 2 min exposure to low-pressure oxygen plasma and baking overnight at 50 °C. The channels were treated to be hydrophobic by Aquapel for 5 min and rinsed with isopropyl alcohol (IPA). A fused silica glass capillary (150 μm O.D., 50 μm I.D.) was directly inserted into PDMS junctions in-line with outlet and inlet channels. Fused silica glass capillaries internal surfaces were treated with Aquapel for 5 min and rinsed with IPA. IPA residue and air bubbles were removed during flushing of all microfluidics device channels with 1-octanol. Every experiment was done with new device and related materials to avoid chemical cross-contamination and allow demonstration of repeatability of measurements.

### 2.3. Microfluidic droplet generation

A T-junction geometry of microfluidic device channels was used to produce picoliter droplets. The aqueous phase contained different concentrations of ACh and GABA dissolved in milliQ water or aCSF. 1-octanol was used as carrier oil phase. The inlets of fused silica capillaries were connected to 100 μL syringes through plastic tubing interfaces. Two syringe pumps (Pump 11-Pico Plus Elite, Harvard Apparatus, Holliston, MA) controlled syringe content displacement creating variable flows of aqueous and oil phases. Found experimentally, the optimal flow rates of aqueous (50 nL/min) and oil (150 nL/min) phases allowing generation of required volume droplets.

### 2.4. Droplet analysis with nESI-MS

The generated droplets moving through fused silica glass capillary (150 μm O.D., 50 μm I.D.) were transferred to Pt-coated, fused silica 30 μm I.D. nESI emitter (PicoTip FS360-50-30, New Objective, Woburn, MA) via a zero-dead-volume Picoclear union (New Objective, Woburn, MA) as it has been reported previously [22]. Mass spectra were acquired using micrOTOF Q-TOF instrument (Bruker Daltonics, Bremen, Germany). Scans were performed at 50 Hz frequency in a positive mode. High mass resolution Bruker MaXis 4G mass spectrometer was used for the quantitative measurements of different concentrations of ACh in aCSF and the analysis of rat CSF samples. The microfluidic PDMS device was mounted on micromanipulator allowing positioning of nESI emitter’s tip at 2 mm distance to orifice of mass spectrometer’s inlet. nESI emitter was grounded by copper tape to connect the Pt coating. For optical monitoring and recording of the droplet sequences inside of the nESI emitter as well as the analysis of the electrospray plume of aqueous and oil phases, a handheld digital microscope (Dinolite, Torrance, CA) was placed above the emitter and green laser was positioned below of the emitter.

### 2.5. Cerebrospinal fluid collection and sample preparation

CSF was harvested from three 2 months old male Sprague-Dawley rats (ENVIGO, Indianapolis, IN), maintained on a 12-h light/dark cycle and fed normal chow ad libitum. Euthanasia using carbon dioxide asphyxiation at conditions reducing animal suffering was performed in compliance with local and federal regulations and according to animal use protocols approved by the Institutional Animal Care and Use Committee (IACUC) at the University of Illinois. CSF was collected directly from the cisterna magna immediately after euthanasia using approach described previosuly [40]. Blood in the CSF samples was not visually observed. CSF samples were frozen on dry ice and stored at −80 °C until the analysis. The CSF samples were diluted with an equal volume of 2 μM ACh-d4 standard solution (prepared in aCSF) which was added into each sample as the internal standard for quantitative analysis. The final concentration of ACh-d4 in each CSF sample was 1 μM.

### 2.6. Statistical analysis

Most of the experiments were repeated at least 3 times. Means and standard deviations were calculated. Linear curve fitting was performed for quantitative measurements. One-way ANOVA was performed on some data sets.

## 3. Results and discussions

### 3.1. Construction and optimization of ultra-small volume droplet microfluidics with nESI detection system

**Fig. 1.**
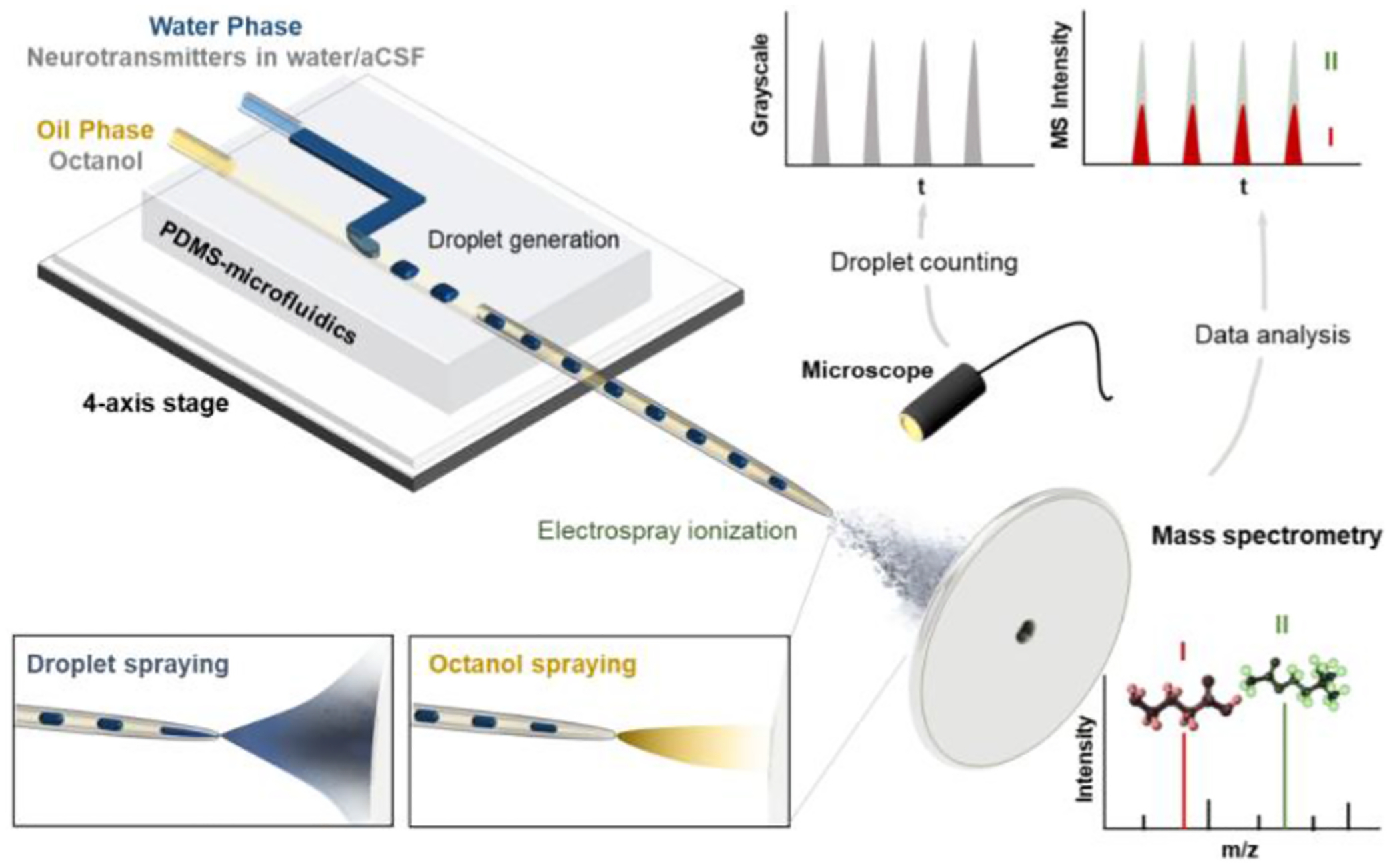
Schematic of droplet microfluidic with MS detection system as well as workflow for optical recording and subsequent nESI-MS analysis of picoliter aqueous droplets containing neurotransmitters segmented by 1-octanol.

One goal was the repeatable generation and MS analysis of picoliter aqueous droplets. To achieve this goal, we designed and tested PDMS microfluidic devices allowing oil-segmented droplets generation by pumping sample (neurotransmitters dissolved in DI water or aCSF) and oil (1-octanol) into two separate arms of a T-junction (Fig. 1). The width of 90° T-junction channel for aqueous phase was 30 μm and for oil phase 50 μm. To prevent generated aqueous droplets from sticking to the surfaces of the PDMS-based T-junction microfluidic devices, outlet capillaries and nESI emitters were treated with Aquapel, a commercially available hydrophobic material. Generated droplets were transferred to Pt-coated, fused silica nESI emitter via a Picoclear zero-dead-volume union (Fig. S1A). A filtration tank (Fig. S1B) is designed for the aqueous phase and oil phase injection ports to block possible impurities in the water and oil as well as to prevent clogging of the ESI tip.

**Fig. 2.**
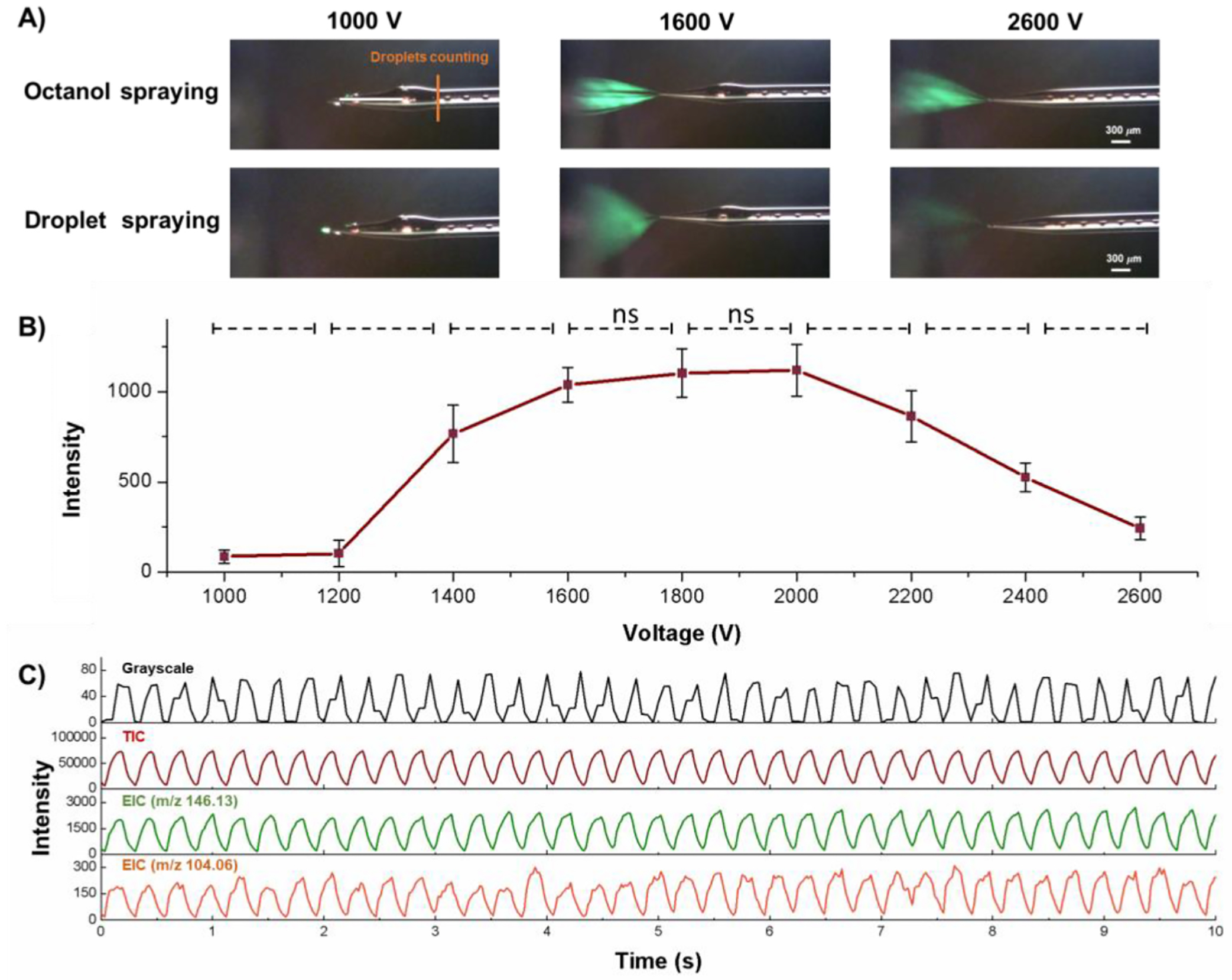
Electrospray voltage optimization for neurotransmitter measurements using the 1-octanol-assisted droplet microfluidics with MS detection. (A) Representative single frames of continuous video recording obtained during electrospraying at voltages 1.0 kV, 1.6 kV, and 2.6 kV. Images of the electrospray emitter’s tip and the surrounding space during oil (1-octanol) accumulation on the emitter (1st image in top row) and electrospray (2nd and 3rd images in top row) are shown. The bottom row images depict aqueous droplet is emerging (1st image in the bottom row) or electrosprayed (2nd and 3rd images in the bottom row). (B) Dependence of intensity for monoprotonated ACh signal on electrospray voltage in arbitrary units. Droplets contained 1 μM ACh. Electrospray voltages are raised stepwise from 1.0 to 2.6 kV in 200 V increments during analysis. (C) Representative traces of greyscale optical recording, total ion current (TIC), extracted ion current (EIC) at *m/z* 146.13 (ACh), and EIC at *m/z* 104.06 (GABA) acquired during mass spectrometric analysis of oil segmented aqueous droplets containing 1 μM GABA and 1 μM ACh. Electrospray voltage was 1.6 kV. Mass spectra were acquired using micrOTOF Q-TOF instrument. Grayscale data are obtained from optical recordings of the aqueous droplets passing through the cross section of the capillary located close to its electrospray emitter’s tip (marked by yellow line on top left image in (A)). MS scan rate in positive mode is 50 Hz. The symbol * indicates significance with p-value <0.05 and n.s. indicates not significant, respectively (one-way ANOVA).

Neurotransmitters, GABA and ACh, were selected as test compounds to evaluate the performance of the system. In the first set of experiments, 1 μM ACh and 1 μM GABA were prepared in DI water. 65 pL droplets were produced at aqueous phase flow rate 50 nL/min and oil phase flow rate 150 nL/min (Fig. S2). Electrospray voltages between 1.0 kV and 2.6 kV are tested. Data were acquired in full scan mode. At voltages less than 1.2 kV, a low signal (less than 100 a.u.) for ACh at *m*/*z* 146.13 was observed (Fig. 2B). At this voltage range, neither the aqueous samples nor the oil plugs generated visible electrospray plumes (Fig. 2A, left). Flat liquid droplets could be seen hanging on the emitter (Fig. 2A, left, bottom) and no analyte’s signal detected. When the voltage was increased above 1.6 kV, a wide fan-shaped spray plume was observed when aqueous droplet was emerging at the tip (Fig. 2A (middle) and ACh signal was detected. However, when the oil plug reached the tip, ACh signal disappeared despite presence of a narrow fan-shaped spray plume.

Analysis of light intensity values of selected pixel on grayscale video recordings acquired by the Dino-lite digital microscope provide information on the size and frequency of aqueous droplets passing through a specific cross-section of the capillary (Fig. 2A). In addition, the video recordings were analyzed at a specific cross section of the spray plume (Fig. S3A). Time sequences of stacked image slices are shown in Figs. S3B and S3C. The timing of the sequence of spray plumes formed by aqueous droplets is close to timing of sequence of droplets observed inside of nESI emitter and was opposite to timing of spray plume formed by oil plugs (Fig. S3D). Fig. 2C compares droplet frequency assessed using video recording and frequencies of detection of signals related to aqueous droplets and presented as the TIC, the EIC of ACh (*m/z* 146.13), and of GABA (*m/z* 104.06) relative signal intensities. The appearance of well-resolved peaks in the TIC and EICs is consistent with sequences of signals obtained using video recording. Performance of our microfluidic system with nESI-MS detection allows analyzing the content of every generated droplet. During the time frames similar to shown in Fig. 2C, the relative standard deviation (RSD) of TIC peak amplitudes was 2 %, and the RSD of EIC at *m/z* 146.13 and *m/z* 104.06 peak amplitudes were 7.2 % and 14.0 %, respectively. Such results demonstrate the system stability for high-throughput nESI-MS analysis. Considering the stability and intensity of the signal, the ESI voltage was set at 1.6 kV for further measurements.

### 3.2. Evaluation of analytical system performances for ultra-small droplets

After the aqueous droplet arrives at the emitter tip and is electrosprayed, peaks are observed in the TICs and EICs. The signals declined to almost zero when the carrier oil phase electrosprayed from the emitter. Figs. S4B–D displays the mass spectra acquired at different time points of the TIC peak (points 1, 2 and 3 on Fig. S4A). Intensities of the observed mass spectra peaks declined when 1-octanol was being electrosprayed, but no new peaks appeared. Thus, despite the observation of 1-octanol-related spray plumes, targeted as well as other cations in observed *m*/*z* range are not detected, demonstrating absence of interference of 1-octanol with MS recording. Interestingly, mass spectrometers with electron ionization are capable to detect a variety of 1-octanol-related cations [41,42]. However, no signals were observed in our measurements. Since PDMS polymers generate some background signals (Fig. S5), additional use of a high-resolution mass spectrometer and/or blank measurements is required for determination if these signals overlap.

Besides 1-octanol, in some experiments PFD was used as the oil phase under the same experimental conditions. The size of the droplets generated and the corresponding TIC are shown in Fig. S6. The results show that the size of the droplets and the recorded TIC are not as stable as those recorded in 1-octanol-based experiments.

**Fig. 3.**
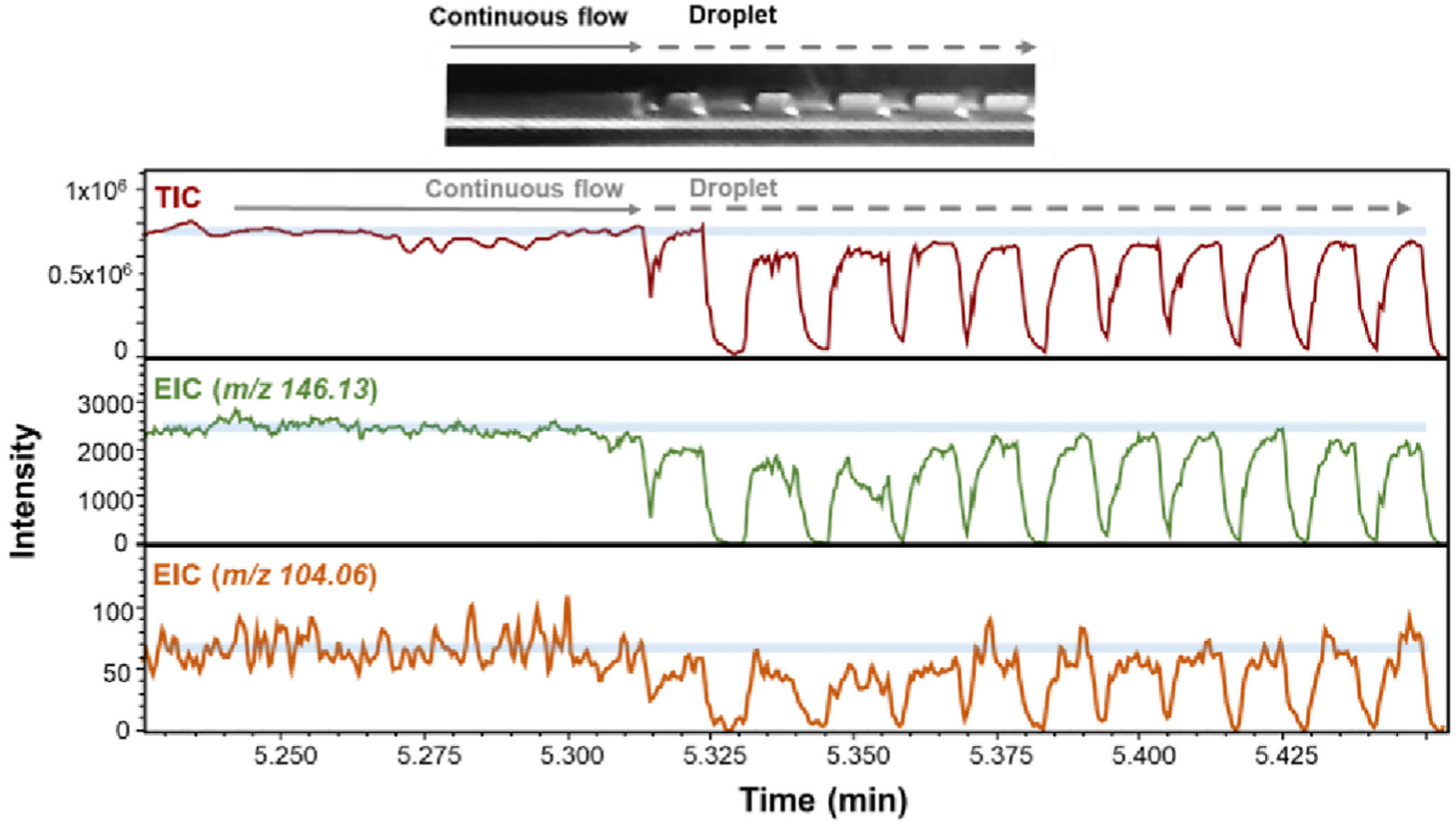
Example of the droplet microfluidics with mass spectrometric detection performance during transition from continuous aqueous phase flow to droplet segmentation. Traces are for TIC, EIC at *m/z* 146.13 (ACh), and EIC at *m/z* 104.06 (GABA). Picoliter droplets (the first droplet ~65 pL) contained 1 μM GABA and 1 μM ACh are formed and analyzed. Top inset shows greyscale image of the moment of transition from continuous flow to droplet segmentation.

We also examined how fast the analytical system responds to switching from a continuous flow of aqueous solution to a segmented droplets flow (Fig. 3). A 200 nL/min flow of containing 1 μM ACh and 1 μM GABA aqueous phase was switched to stream of picoliter aqueous droplets separated by 1-octanol plugs by injecting the oil phase into T-junction. After switching, the ACh- and GABA-containing droplets were generated at T-junction with 50 nL/min flow of aqueous phase and 150 nL/min flow of oil phase in corresponding channels of the microfluidic device. 5.3 min after switching, droplets are optically detected at the nESI emitter. As shown in Fig. 3, the comparison of TIC, ACh EIC, and GABA EIC with optical signals demonstrated theirs good temporal match. Immediately after switching, generated droplet sizes are not uniform, since the microfluidic chip needs time to stabilize forces involved in droplet generation. The volume of the first aqueous droplet is optically determined and calculated to be ~65 pL. Importantly, comparison of the signal intensities (e.g. GABA) for MS recordings before and after switching from continuous aqueous flow to droplet-segmented sample delivery demonstrates only a small decrease in these signal intensities. This indicates that 1-octanol as well as segmented flow/electrospray have little effect on the detection of ACh and GABA in our setup.

**Fig. 4.**
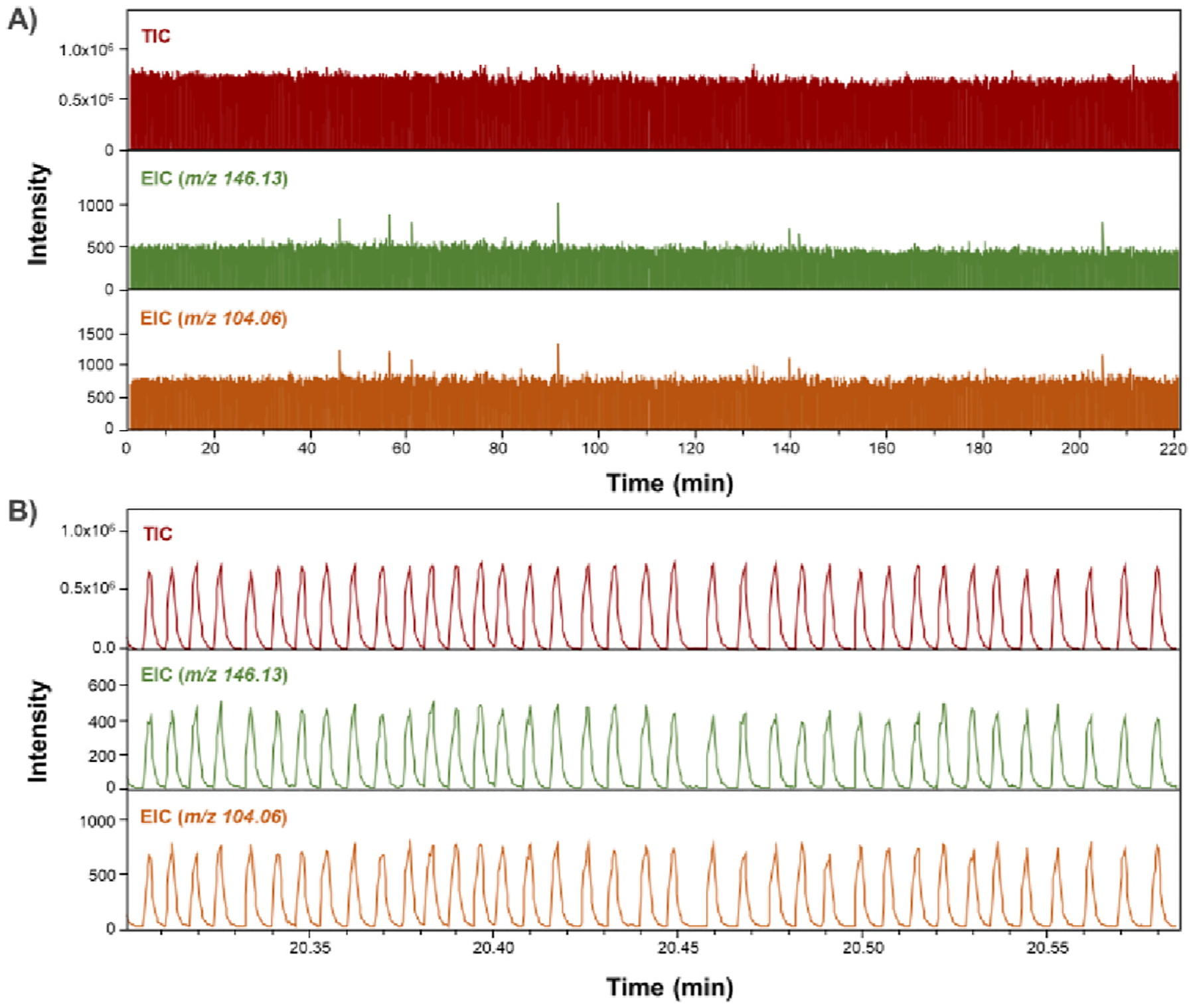
Temporal stability of droplet microfluidic system with MS detection. (A) Results of analysis of 65 pL droplet stream for 220 min. TIC, EIC at *m/z* 146.13 (ACh) and EIC at *m/z* 104.06 (GABA) during 220 min of analysis are shown. Droplets contained 1 μM GABA and 1 μM ACh. (B) A zoomed range of recordings depicted in (A).

We evaluated the long-term stability of droplet generation and analyte detection by nESI-MS. In these experiments, 65 pL droplets, containing 1 μM GABA and 1 μM ACh, are generated. Continuous MS analysis of droplets was performed for 220 min. More than 25,000 droplets are generated and their content measured (Fig. 4A). The established system enabled high-throughput detection of approximately 113 droplets per minute or 1.9 droplets per second. Fig. 4B shows stable detection of analytes in generated droplets with no interference between individual droplet samples.

Droplet microfluidics provides both digital sampling and analysis, but it also allows off-line sample collection and storage. As this is one of our goals, we evaluated stability of aqueous droplets segmented by 1-octanol inside of fused silica capillary (Fig. S7). The tested conditions allowed observation of intact aqueous droplets for up to 12 days. This demonstrates that the developed system can be used for sample storage and remote sample collection.

### 3.3. Quantitative analysis of neurotransmitters in CSF samples

**Fig. 5.**
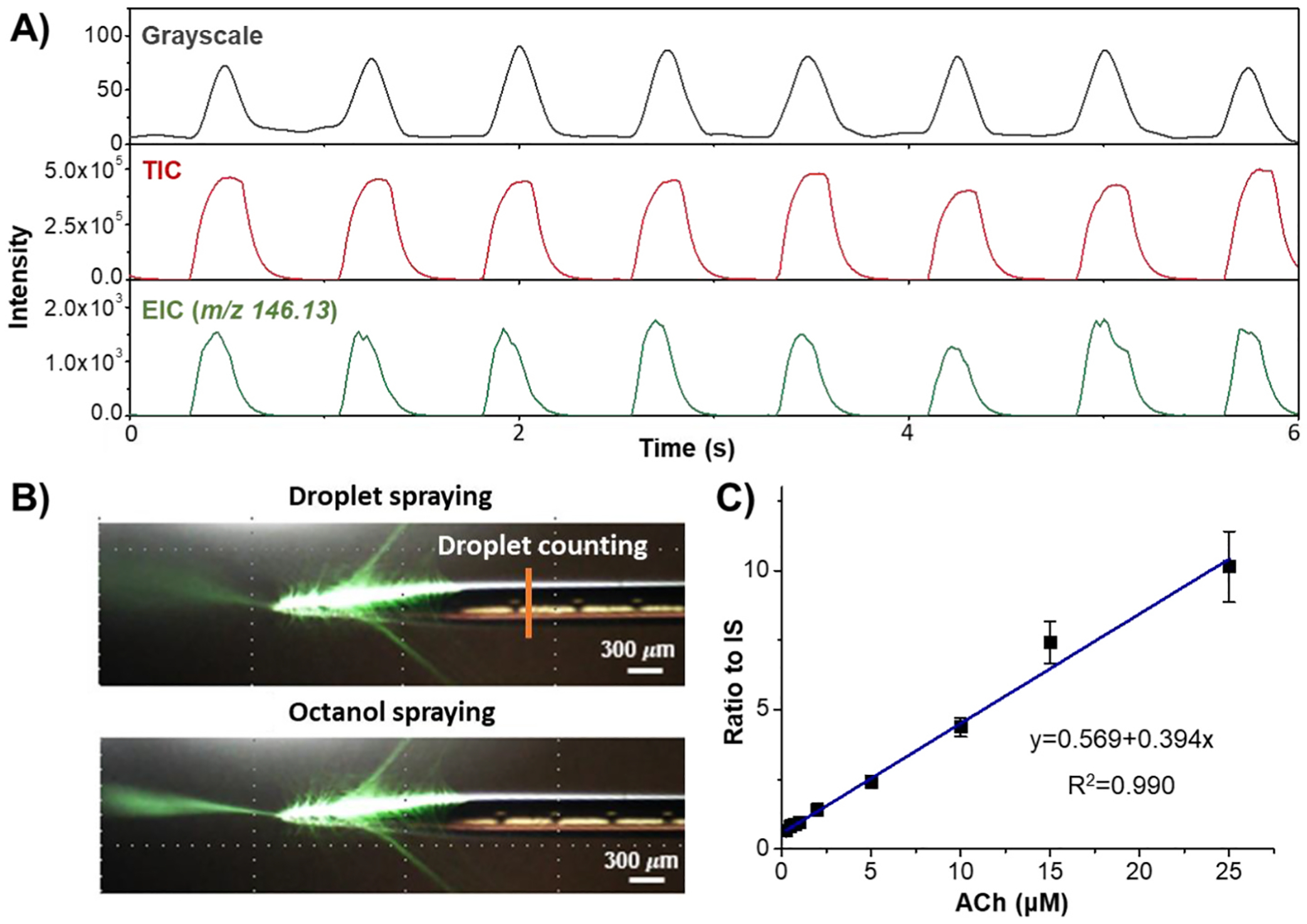
Performance of the droplet microfluidics sampling system with MS detection for measuring of neurotransmitter in aCSF droplets. (A) Grayscale optical recording (upper trace), TIC (middle trace) and ACh EIC at *m/z* 146.13 (lower trace) obtained during oil segmented aCSF droplet motion through the electrospray emitter and MS detection. Droplets contained 1 mM ACh. The droplet frequency is 1.33 Hz and the stream speed is 0.5 mm/s. The distance between the optical recordings site and emitter’s tip end is 2 mm. Therefore, the aqueous droplet detection at the vertical line in emitter and the sequence of oil spray plumes and droplet spray plumes are synchronized with 4 s delay. Therefore, the optical trace was manually realigned with the EIC and TIC traces. (B) Images of single frames of continuous video recording captured during electrospray of aqueous droplet (upper image) and oil plug (lower image) from the electrospray emitter’s tip. Measurements of the aqueous droplets is performed using converted to grayscale optical recordings at the cross section of the capillary located close to its electrospray emitter and marked by vertical yellow line on the top image. (C) Concentration dependence of ACh detection in aCSF. Plot of the ACh signal intensity normalized to signal intensity of stable isotope labeled internal standard (IS) versus ACh concentrations (n = 10, R^2^ = 0.99).

To test the analytical system performances with more chemically complex samples, ACh was dissolved in artificial cerebrospinal fluid to approximate our overarching goal of *in vivo* monitoring of neurotransmitters from brain slices and from the brain of live animals. The same experimental conditions were used. Stable 65 pL aCSF droplets containing 1 mM ACh are generated (Fig. 5A). The shapes of spray plumes in electrospray are also similar to ones observed with DI water droplets (Fig. 5B). Even though aCSF contains high concentrations of inorganic salts, the performance of the droplet microfluidic system remained unchanged. To determine how well the system works for quantitative analysis, aCSF droplets containing varying concentrations of ACh and the internal standard, 1 μM ACh-d4, were examined. Evaluation of detected ACh signals normalized to internal standard signals demonstrated concentration-dependent linear response (R^2^ = 0.99) in range of concentrations spanning between 0 and 25 μM and results from measurements of the analyte in 10 aCSF droplets for each concentration are summarized in Fig. 5C (also Figs. S8 and S9). These measurements demonstrated stability of pL droplet generation and sufficient sensitivity of analyte detection in presence of relatively high inorganic salt content. As a final example, we measured ACh in three CSF samples collected from three individual rats. 1 μM ACh-d4 was added into each sample as the internal standard for quantitation analysis. The endogenous concentrations of ACh in CSF were determined to be 0.89 ± 0.09 μM, 0.95 ± 0.08 μM, 1.15 ± 0.09 μM (mean ± SEM, n = 3 animals; data acquired from 10 CSF droplets for each animal were summarized). The limit of detection (LOD) was calculated as 3 × standard deviation of blank/slope. The LOD for ACh was calculated to be 370 nM for 65 pL droplets, which corresponds to 24 amol. Our data calculated slightly higher concentrations of ACh in CSF than previously reported [43,44] which may occur due to differences in sampling location, animals, or and distinct CSF sample collection protocols. A limitation of 1-octanol as an oil phase is that it is most suitable for detecting hydrophilic analytes, as hydrophobic substances may be soluble in 1-octanol.

## 4. Conclusions

In summary, a microfluidic system was developed for detection of neurotransmitters in ultra-small volume droplets. 1-octanol used as the carrier oil allows the robust generation of aqueous droplets as small as 65 pL in volume. Our developed analytical system demonstrated high sensitivity, reliability, and throughput of analysis. The successful application of nESI-MS to biological pico-liter droplet samples demonstrates the potential of our method for other droplet-microfluidics-based work, such as rapid biological fluid analysis, monitoring cellular secretions, and evaluating of spatiotemporal enzymatic activity. The established system will be applied to the analysis of different microenvironments at high spatiotemporal resolution, and has additional application to *in vivo* monitoring of neurochemicals and infused drugs in the brain of live animals.

## Supplementary Material

Supplementary

## Data Availability

Data will be made available on request.

## HIGHLIGHTS

- A pL droplet microfluidic-nESI MS system optimized for neurotransmitter detection.
- The 1-octanol oil phase effectively generates aqueous droplets as small as 65 pL.
- Long-term stability for droplet generation and analyte detection by MS.
- Acetylcholine quantified from rat cerebral spinal fluid samples.
- Characteristics of the system designed for *in vivo* monitoring of neurotransmitters.
