# Supplementary material for: 1-Octanol-assisted ultra-small volume droplet microfluidics with nanoelectrospray ionization mass spectrometry
Source: Anal Chim Acta. Author manuscript; Available in PMC 2024 Sep 20. (PMC11413884; doi:10.1016/j.aca.2024.342998)
Supplement: Supplementary [file NIHMS2022706-supplement-Supplementary.pdf]

Supporting Information

1-octanol-assisted ultra-small volume droplet microfluidics with nanoelectrospray ionization mass spectrometry

Yaoyao Zhao,[#] Insu Park,[#] Stanislav S. Rubakhin, Rashid Bashir, Yurii Vlasov, and Jonathan V. Sweedler*

Y.Z. and I.P. are co-first authors with equal contributions to the work.

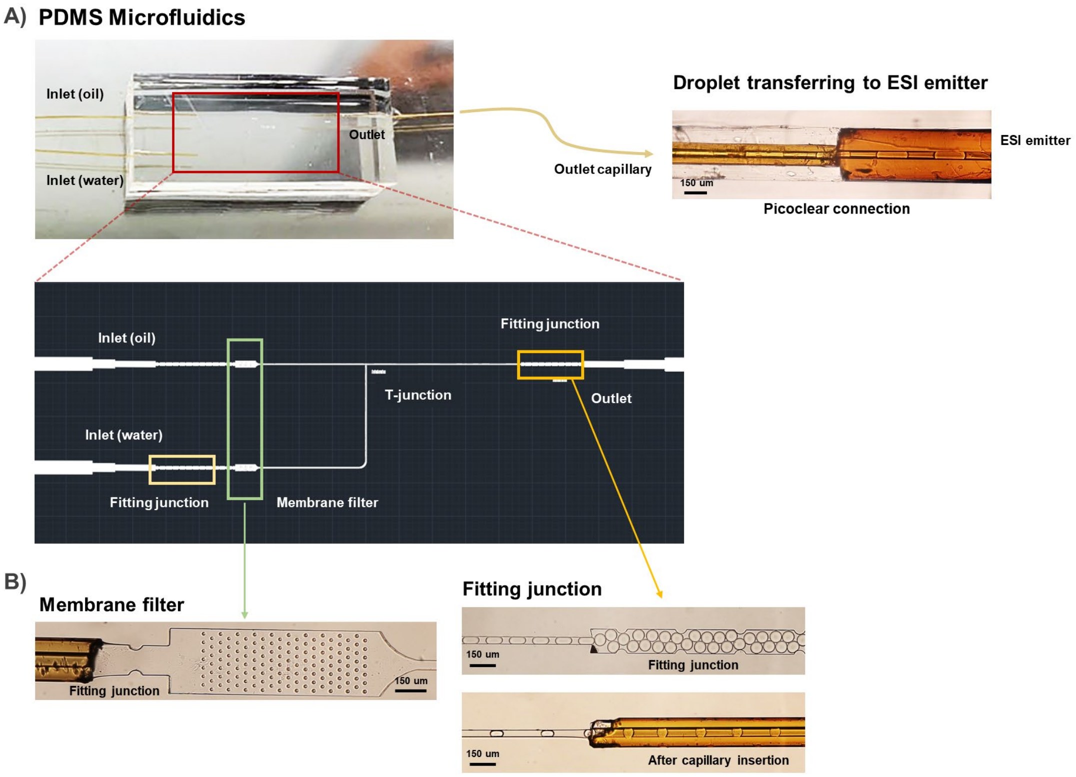

Figure S1. Optical images of PDMS-based microfluidics and fitting junctions for transferring pico-liter volume droplet to nESI emitter. (**A**) PDMS-based microfluidics with two inlets and one outlet connected to fused silica capillaries. Pico-liter droplet was transferred to silica capillary and then to nESI emitter through a Picoclear zero-dead-volume union connector. (**B**) Microphotographs depicting membrane filtration units placed in line with aqueous phase and oil phase channels to avoid the channel and nESI emitter clogging by particles. Serration-like fitting junction designed to avoid a solution leakage between capillary and PDMS-based device surfaces.

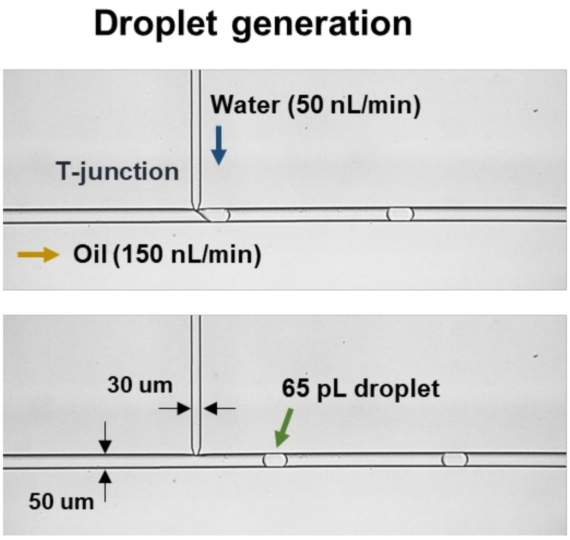

Figure S2. T-junction based microfluidics for pico-liter droplet generation. Water or aCSF were used as water phase and 1-octanol was used as oil phase. 65 pL droplets were produced at aqueous phase (sample) 50 nL/min flow rate and oil 150 nL/min flow rate.

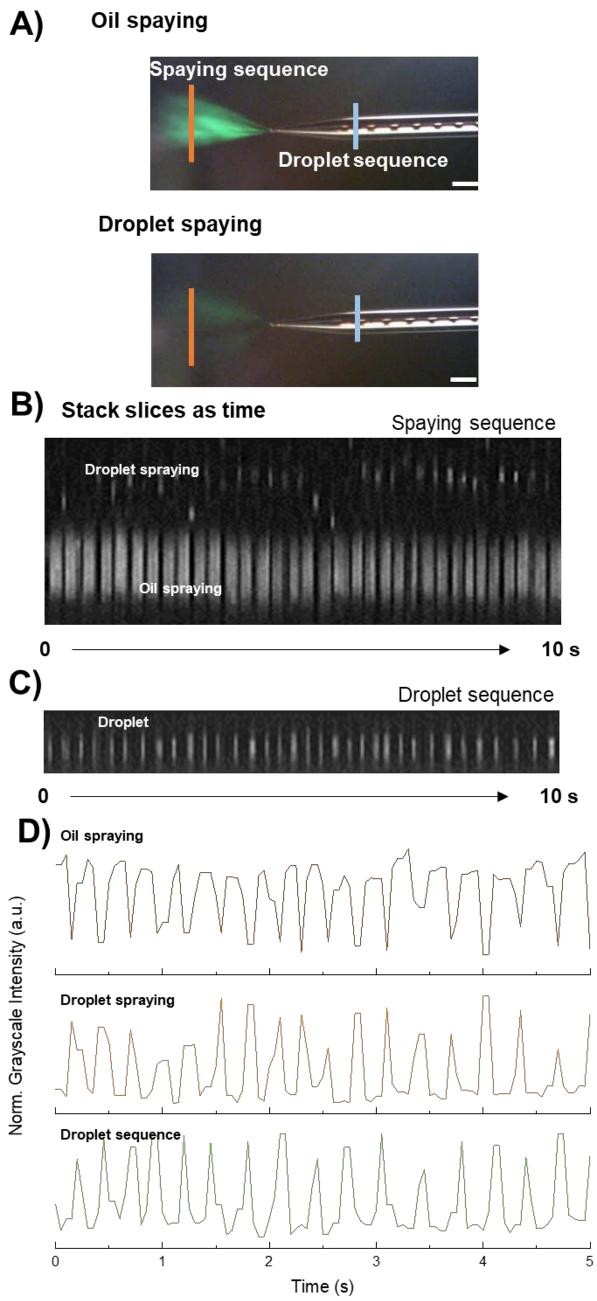

Figure S3. Characterization of aqueous droplet and oil plug sequences and their electrospraying patterns using time-lapsed image analysis. (**A**) Optical images of droplet and oil plug electrospraying. Vertical lines show locations for data acquisition. Scale bar is 300 um (**B**) Continuous sequence of line scans of spray plumes (bright regions) of electrosprayed aqueous droplets and oil plugs (orange line in Fig. S3A). The areas of bright regions representing oil plug sequences are larger than that of aqueous droplet sequences are likely due to difference in volumes, density, and size of formed. Image J software was used to extract and stack slices as well as measure grayscale intensity values from time-lapsed images. (**C**) Continuous stacked

slices of aqueous droplet sequences detected inside of nESI emitter (light blue line in Fig S3A). (**D**) Relative grayscale signal intensity values determined at spray plume locations for electrospraying of oil plugs (top trace), aqueous droplets (middle trace) and aqueous droplet sequences inside of nESI emitter. The time aligned spray plumes aqueous droplet sequence is similar to the sequence of droplets inside of nESI emitter. Timing in the sequence of spray plume of oil plug spraying is alternated with the timing of spray plume of aqueous droplet spraying. The droplet frequency is 3.4 droplets/second and the droplet velocity inside of the capillary is 3.35 mm/second. The length between the vertical line of droplet sequence (light blue line in Fig. S3 (A)) and tip end is 1 mm. Therefore, the aqueous droplet detection at the vertical line in emitter and the sequence of spray plume of oil and droplet sprayings are synchronized with 0.3 seconds delaying. The time delay is not a fixed value for each experiment, which needs to be recalculated if the position of microscope is changed.

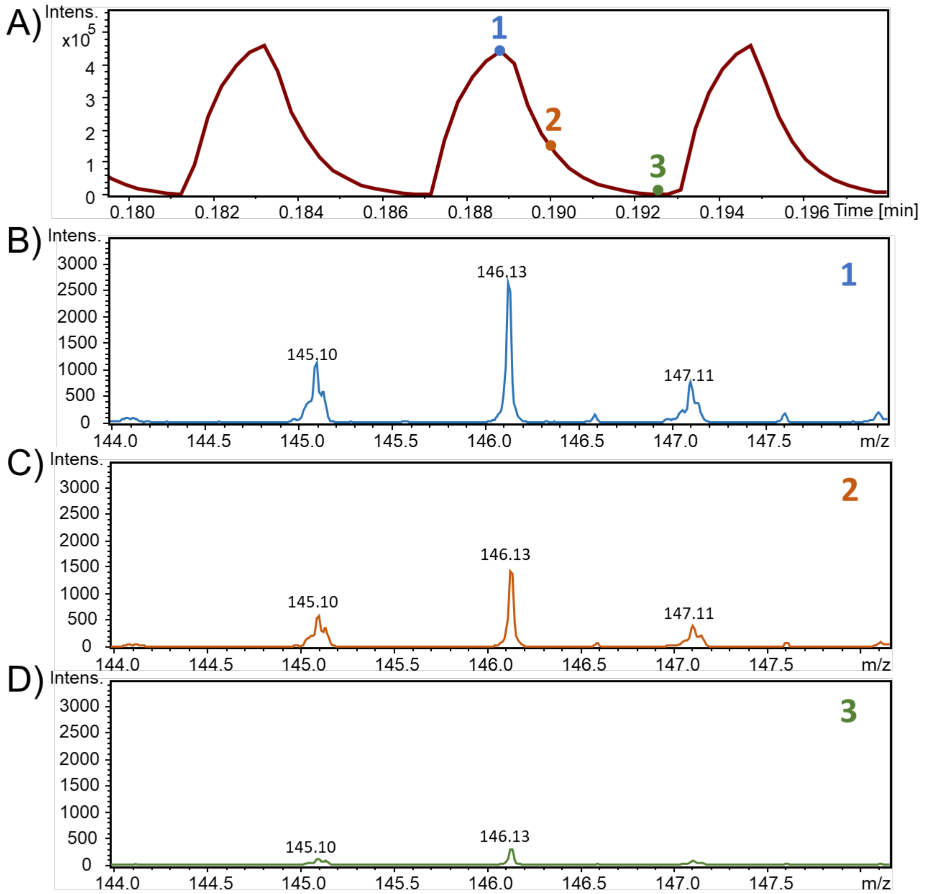

Figure S4. (A) TIC corresponding to detection of different ions including ACh in individual aCSF droplets. (**B**), (**C**), and (**D**) show mass spectra acquired at time points 1, 2, and 3 marked on TIC trace in (**A**), respectively. Mass spectra acquired using micrOTOF Q-TOF mass spectrometer (Bruker). Data were acquired in positive mode with scan rate set at 50 Hz.

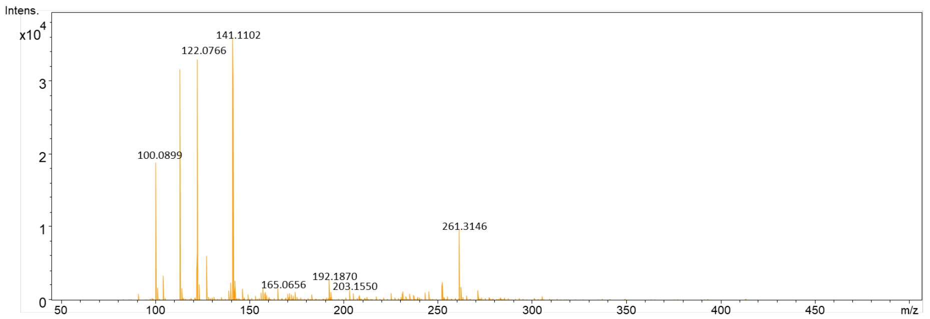

Figure S5. The mass spectrum of blank sample containing pure water. The mass spectrum was obtained using entire assembled system containing a PDMS chip, connective lines, and nESI-MS system.

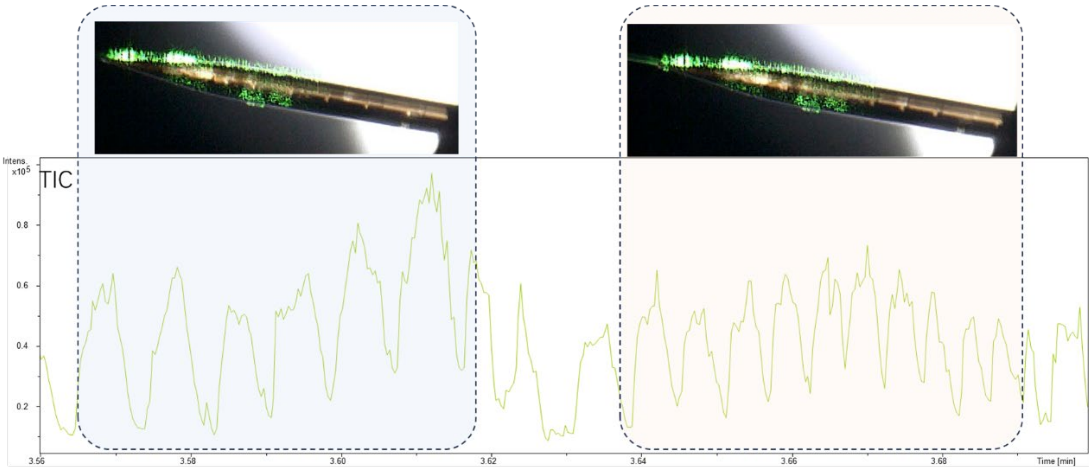

Figure S6. Representative TICs and optical images obtained using developed microfluidic setup and PFD as the oil phase

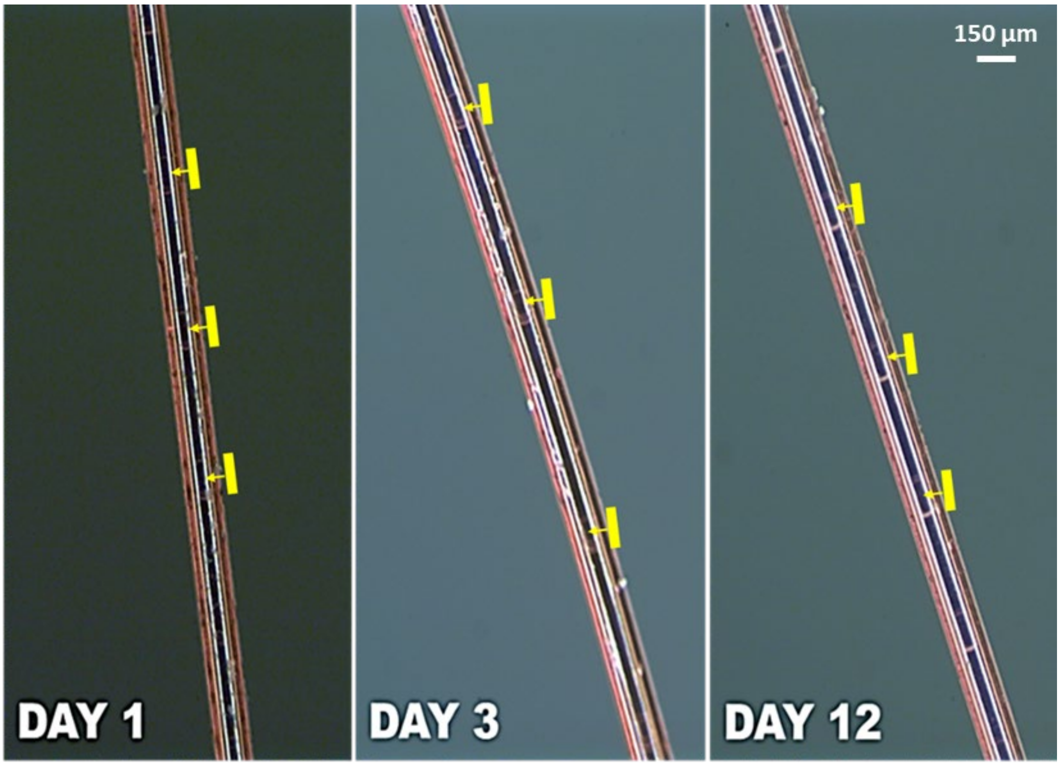

Figure S7. The morphology of the aqueous droplets in the fused silica capillary on the first day, the third day, and the 12th day of storage at ambient conditions. Locations of droplets are marked by arrows and rectangles.

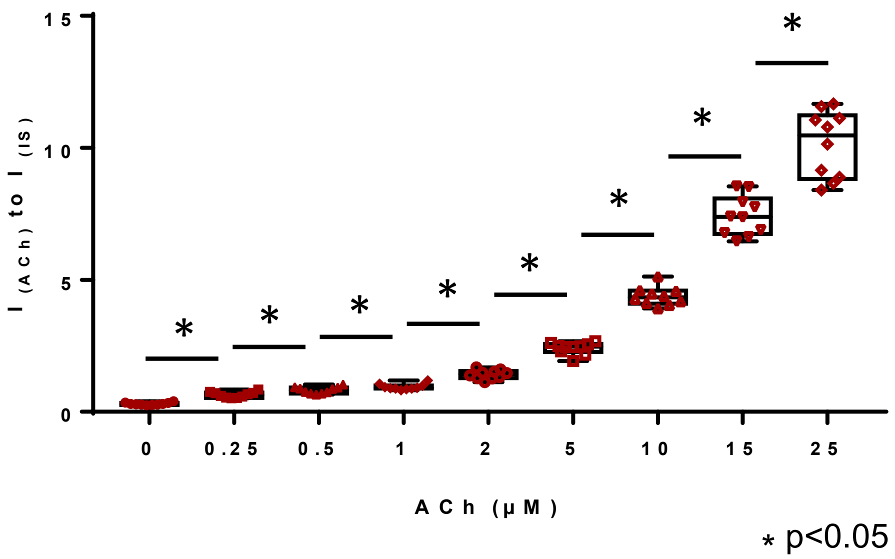

Figure S8. Concentration dependence of ACh detection in aCSF.

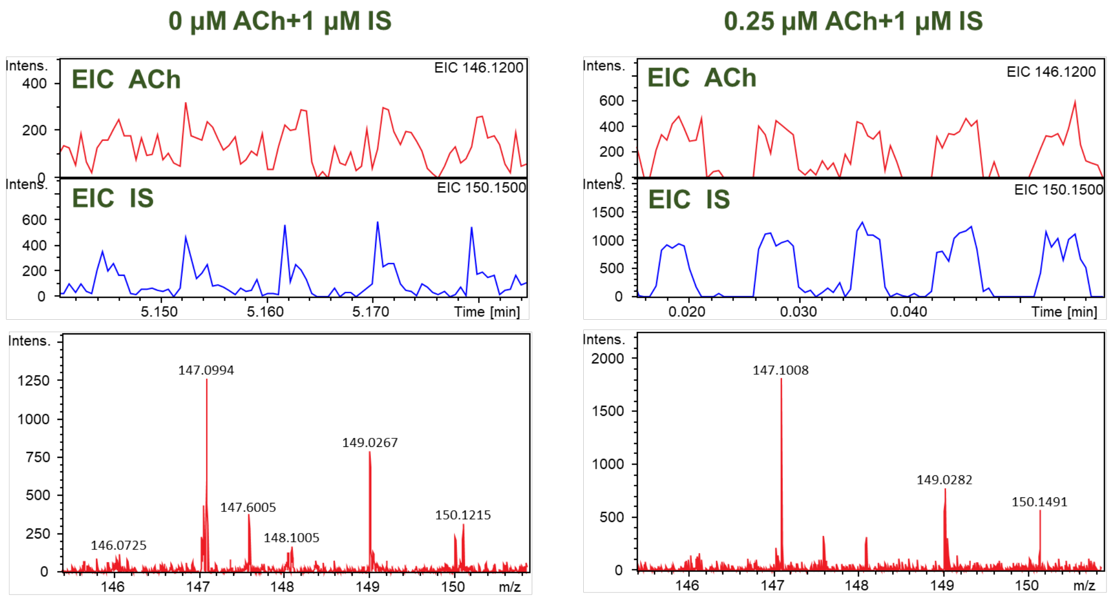

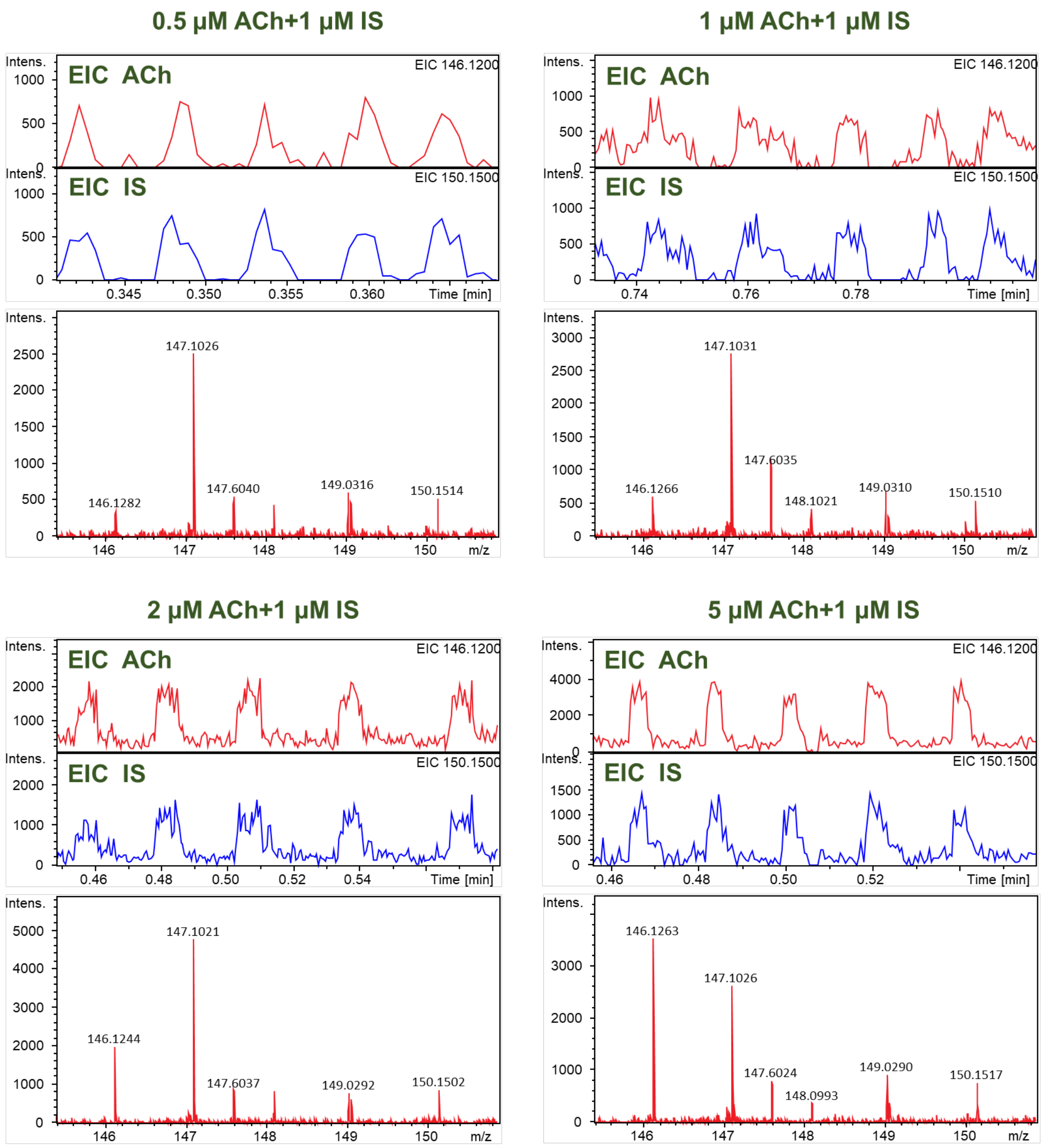
0.5 µM ACh+1 µM IS
1 µM ACh+1 µM IS
2 µM ACh+1 µM IS
5 µM ACh+1 µM IS
EIC ACh
EIC IS
EIC 146.1200
EIC 150.1500
Intens.
Time [min]
m/z
147.1026
146.1282
147.6040
149.0316
150.1514
147.1031
147.6035
146.1266
148.1021
149.0310
150.1510
147.1021
146.1244
147.6037
149.0292
150.1502
146.1263
147.1026
147.6024
148.0993
149.0290
150.1517

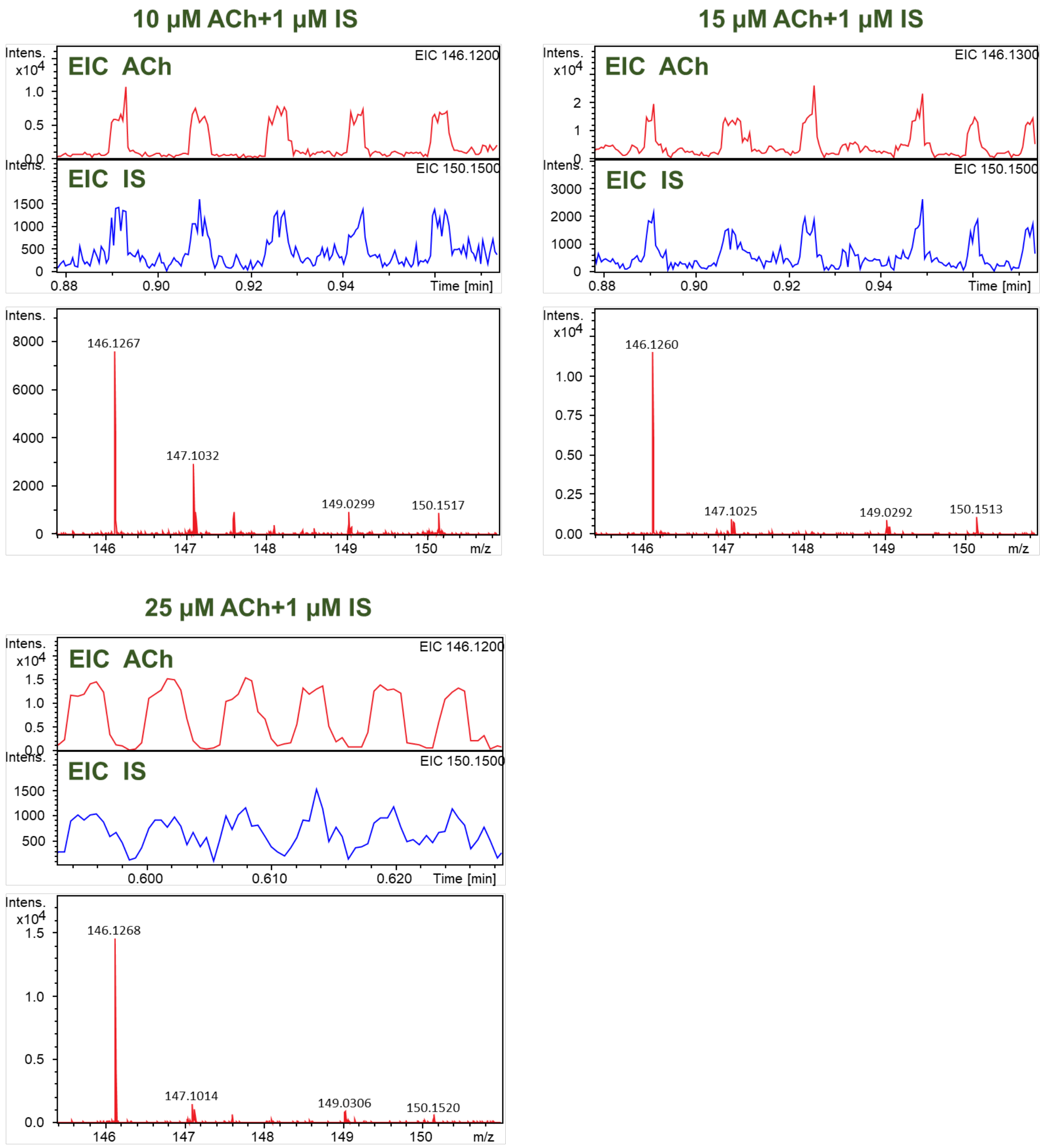

Figure S9. Examples of EICs for ACh and IS as well as representative mass spectra depicting detected ACh signal used in determination of LOD of the developed approach.
